# Supplementary material for: Dual neutrophil subsets exacerbate or suppress inflammation in tuberculosis via IL-1β or PD-L1
Source: Life Sci Alliance. 2024 May 21;7(7):e202402623. doi: 10.26508/lsa.202402623 (PMC11109925; doi:10.26508/lsa.202402623)
Supplement: Supplementary file 2 [file LSA-2024-02623_TableS2.docx]

| **Gene** | **Forward** | **Reverse** | **Gene** | **Forward** | **Reverse** |
| --- | --- | --- | --- | --- | --- |
| *Adgre1* | CAGCCAGAGGGGACAAGATGA | TCCTGGGGCCCCTGTAGAT | *Il18rap* | CCAGTCTCAGCTGCCAAAGT | AGGAAGTAGTCTTCCATCCTTGTA |
| *Aim2* | TGGGTGGCGTCAGGAAGTTT | GGCCGGTCAACAACAGCATTT | *Il1a* | CGCTTGAGTCGGCAAAGAAATCA | TGCAAGTCTCATGAAGTGAGCCA |
| *Arg1* | TAGAGAAAGGCCCTGCAGCA | TCAACAAAGGCCAGGTCCCC | *Il1b* | TCTAATGCCTTCCCCAGGGC | GACCTGTCTTGGCCGAGGAC |
| *Atg7* | CCTGACCTTCGCGGACCTAA | GCCTTCGGCTCGACACAGAT | *Il1r1* | ACCGTGAACAACACAAATGGAGAA | ATGGTGTCGCCGTGCATTTT |
| *Casp1* | GGCATGCCGTGGAGAGAAAC | TGGGCCTTCTTAATGCCATCATC | *Il1r2* | GGAGACCCCACACGCCTATT | GGGTTCCGTGGTTGTTCCTTTG |
| *Casp3* | GAGCTTGGAACGGTACGCTAAG | GTCCACTGACTTGCTCCCATGTA | *Il1rn* | GGAAGACCTTGTGTCCTGTTTAG | GGCACCATGTCTATCTTTTCTTC |
| *Casp4* | TCCCAGATGCCCACCATTGA | TCAGTTGCTTGTTGCTTTGTTCTC | *Il33* | GCTGCGTCTGTTGACACATT | GACTTGCAGGACAGGGAGAC |
| *Casp8* | CCACAGACCCCAGACAGAGAA | AGAGGTAGAAGAGCTGTAACCTTAT | *Il4* | ACGGAGATGGATGTGCCAAACGTC | AACTTTCCAGGAAGTCTTTCAG |
| *Cat* | AGATTGCCTTCTCCGGGTGG | CCCGACTGTCCGACATGGTG | *Il6* | GAGGATACCACTCCCAACAGACC | AAGTGCATCATCGTTGTTCATACA |
| *Ccl20* | CTTCCTTCCAGAGCTATTGTGG | TCATCCATTGGACAAGTCCACTG | *Il6ra* | CAACACCACCAACGGGAAGA | GGATCCGGCTGCACCATTTTT |
| *Ccl5* | TCTCTGCAGCTGCCCTCACC | TCTTGAACCCACTTCTTCTC | *Inos2* | GCCACCTTGGTGAAGGGACT | ACGTTCTCCGTTCTCTTGCAGT |
| *Ccl7* | CCTGGGAAGCTGTTATCTTCAA | CCTCCTCGACCCACTTCTGAT | *Irf1* | CCAGCATCTCGGGCATCTTT | GAGTGATTGGCATGGTGGCTTTG |
| *Ccl8* | ACGCTAGCCTTCACTCCAAAATC | GGCTGACAGGGACAGCTATGA | *Irf3* | CAATTCCTCCCCTGGCTAGA | AGATGCCAAAGTCAGCCATCTG |
| *Ccr1* | CTCTGGAAACACAGACTCACTGTC | TTGGCATGGAGTGGAGTCCC | *Irf7* | GGTCCAGCGAGTGCTGTTTG | CACAGCCCAGGCCTTGAAGA |
| *Cd14* | AAGCCCGTGGAACCTGGAAG | CACGCTCCATGGTCGGTAGA | *Irf9* | TCAGGCCCTGCCCATTTCTTC | TTTGCCTGAGGCCATCCTTCT |
| *Cd209a* | ACAGTCAAGTCCCCTTGGCA | AGTCGATCTACGCCAGCCTTC | *Itgam* | GCTCTCATCACTGCTGGCCT | GTTACTGAGGTGGGGCGTCT |
| *Cd28* | TGGCTCTTTGTGTTATCTGGACAAA | GGGCGTAGGGCTGGTAAGG | *Ly6g* | CCCTGCTGTATAGGCACCCC | ATGCCTCCAGGGTCAAGAGC |
| *Cd40* | TCACCATTTTCGGGGTGTTTCT | CCGCAGGGGGTAAGATCTCATT | *MhcII* | GGAGCAAGATGTTGAGCGGC | GCCTCGAGGTCCTTTCTGAC |
| *Cd74* | TGGATGGCGTGAACTGGAAGA | TCCTGGCACTTGGTCAGTACTTT | *Mki67* | CATCATTGACCGCTCCTTTAGGTAT | GGTATCTTGACCTTCCCCATCAG |
| *Cd80* | CGACTCGCAACCACACCATTA | GGAGGGTCTTCTGGGGGTTTT | *Mlkl* | GTCTTTCTGGCAGAGAACGAATCT | TACACCTTCTTGTCCGTGGATTCT |
| *Cd86* | CGCGTAAGAGTGGCTCCTGTA | CCAAGCCCATGGTGCATCT | *Mmp7* | TTTGATGGGCCAGGGAACACTCTA | ATGGGTGGCAGCAAACAGGAAGT |
| *Cgas* | AAGGCTGGCTGGGCACAAA | CGCCAGGTCTCTCCTTGAAAA | *Mmp8* | ACAGGGAACCCAGCACCTATT | TGGGGTTGTCTGAAGGTCCATAG |
| *Clec7a* | GGGGATCAGAGAAAGGAAGCCA | CAGCACTGCAGCAACCACTAC | *MMP9* | ACCACCACCACCACACACAA | CTGCCTCCACTCCTTCCCAG |
| *Col1a1* | GCTACTACCGGGCCGATGAT | CGATCCAGTACTCTCCGCTCTT | *Mpo* | TGTCCGTGTCAAGTGGCTGT | GGGGCTTCGTCTGTTGTTGC |
| *Col3a1* | ACCCAGAGGAGTAGCTGGAGAA | TCCGGGCATACCCCGTATC | *Myd88* | CACTCGCAGTTTGTTGGATG | CGCAGGATACTGGGAAAGTC |
| *Ctla4* | GTCTGTGTGGGTTCAAACACATCT | AGGTCCTCAGGGAGCAGAGTAA | *Nfkb* | CCACGAGGCAGCACATAGATGA | GCAGTGGGCTGTCTCCAGTA |
| *Ctsg* | AATGTGCGCCAATCGCTTCC | GAATCACCCCTGAAGGCAGAC | *Nlrc4* | GACGCTTTGACTCACCACAATGAA | CTGCTCCAAGGGCTCACAGTA |
| *Ctsk* | CCCATATGTGGGCCAGGATGAAA | TTCTCGTTCCCCACAGGAATCT | *Nlrp3* | TGCGTGTTCTCTGTATACCACATCT | GGGCTTAGGTCCACACAGAAAGT |
| *Cxcl1* | CGCTCGCTTCTCTGTGCAGC | GTGGCTATGACTTCGGTTTGG | *Oas1* | GGTCAAGGGCAAAGGCACCA | TCTCATGCTGAACCTCGCACA |
| *Cxcl10* | CACGTGTTGAGATCATTGCCA | GCGTGGCTTCACTCCAGTTA | *Oasl2* | CCCACCAACAACCTGGGAAGA | ACATCCCTCGCTCGCTGTA |
| *Cxcl2* | GCTGCTGGCCACCAACCACC | TGAGAGTGGCTATGACTTCTG | *Orl1* | GGCTGAGGTCCTCGACTGTTTC | GGAAGAAAGCAAATGCAGACCTTTA |
| *Cxcl5* | CCCTACGGTGGAAGTCATAGCTAAA | GCCGTTCTTTCCACTGCGAG | *P2rx7* | CGGATCCAGAGCACGAATTATGG | CGCTCACCAAAGCAAAGCTAATGT |
| *Cxcr1* | CCAGCTGGTGCCTCAGATCAAA | TGGGCAGCATTCCCGTGATA | *Pdcd1* | CTGGAGCAGAGCTCGTGGTAA | AGCTCCTCTGGCCTCTGACATA |
| *Cxcr2* | TCAACCAGCCCTGACAGCTC | ACTTAATCCTGCAGTAGTTCTACGA | *Pdl1* | GCAGGCGTTTACTGCTGCAT | TGCGGTATGGGGCATTGACT |
| *Cxcr3* | CCAAGCCATGTACCTTGAGGTTAGT | AGTCGCTCTCGTTTTCCCCATA | *Ppia* | GCTGGACCAAACACAAACGG | CCAAAGACCACATGCTTGCC |
| *Cybb* | TGGGATGAATCTCAGGCCAATCA | CCAGTTGGGCCGTCCATACA | *Prtn3* | AGCAGGCATATGCTTCGGAGA | CCCCGCAGCACGTTTTGAAT |
| *Ela2* | TCAGCAGCCCACTGTGTGAA | AGAAGGTCTGTCGAGTGCGC | *Ptgs2* | AGCCAGGCAGCAAATCCTTG | ACTGTGTTTGGGGTGGGCTT |
| *Fcgr1* | TGATTCTTACCAGCTTTGGAGATGA | CCACCGACTGGAACCCAAAG | *Rpl4* | GACCAGTGCTGAGTCTTGGG | GTATTCACTCTGCGGTGCCA |
| *Gmcsf* | TGCAGACCCGCCTGAAGATA | GGCCTGGGCTTCCTCATTTT | *S100a8* | TCCTTTGTAAGCTCCGTCTTC | CTTCTCCAGTTGAGACGGCA |
| *Gsdmd* | TCCCGGGTTGAGCAGACAAT | CGATGGCATGGTCCTCGATTT | *S100a9* | GTGGAAGCACAGTTGGCAAC | TGGGTTGTTCTCATGCAGCT |
| *Hprt1* | CAGTCCCAGCGTCGTGATTA | TGGCCTCCCATCTCCTTCAT | *Slc11a1* | TGGCCATTGGGGCTCAGAT | TCCAGCTTGCGCAAACCATAG |
| *Ifi204* | GCTGATTCTGGATTGGGCAAACT | CAGTGATGTTTCTCCTGTTACTTCT | *Socs1* | GACGCCTGCGGCTTCTATTG | GACTGTCGCGCACCAAGAA |
| *Ifna* | CTCCTAGACTCATTCTGCAATGA | GGGCTCTCCAGACTTCTGCTCTG | *Socs2* | CTGGAGCCTCCGGGAATG | TCCCCAGTACCATCCTGTTTGA |
| *Ifnar1* | CTCCCCGCAGTATTGATGAGTTTT | CTCAGGCGCGTGCTTTACTT | *Socs3* | CGCGGGCACCTTTCTTATCC | GGGTCACTCTGCAGCGAAAA |
| *Ifnar2* | CACCGTCTGCTTTTGATGGGTAT | GGTGGGCCAGACTTGTTCTC | *Sod2* | TGAGCCCTAAGGGTGGTGGA | ACGGCTGTCAGCTTCTCCTT |
| *Ifnb1* | AAGCAGCTCCAGCTCCAAGAA | TGGATGGCAAAGGCAGTGTAAC | *Stat1* | TCAAGCTGAGACTGTTGGTGA | TGTGTGCGTACCCAAGATGT |
| *Ifng* | TCTTCTTGGATATCTGGAGGAA | AGCTCATTGAATGCTTGGCGCTG | *Stat2* | AGCATTTGGCTACCTGGATTGA | GCCATTGGGAAAGGTCTGAAT |
| *Ifngr1* | GGTGCCTGTACCGACGAATG | GGTGCCTGTACCGACGAATG | *Stat3* | GCTGCCCCGTACCTGAAGA | TGTCAAACGTGAGCGACTCAAA |
| *Ifnlr1* | GGTGCCTGTACCGACGAATG | CAGTCCAGGAACCCGAATACAC | *Tbk1* | TCAGGCTGGCCACCAGAAA | TCTCTTGGATGCGTGCCTTCT |
| *Il10* | ATGCTGCCTGCTCTTACTGAC | CTGGGGCATCACTTCTACCAG | *Timp1* | TCCTAGAGACACACCAGAGCAGAT | GGGAACCCATGAATTTAGCCCTTAT |
| *Il10rb* | CGAGCCCGCAGCTGTTT | GATCTTGGAAAGACCTGTAACTTTC | *Tlr2* | GCATCCGAATTGCATCACCG | CATCACACACCCCAGAAGCA |
| *IL12p40* | CTCACATCTGCTGCTCCACAA | GACGCCATTCCACATGTCACT | *Tlr4* | TTATCCAGGTGTGAAATTGAAAC | GCCACATTGAGTTTCTTTAAGG |
| *Il17* | TCCAGAAGGCCCTCAGACTA | AGCATCTTCTCGACCCTGAA | *Sting* | GGTCTAGGAAGCAGAAGATGCCATA | TCAGGCTGGCCACCAGAAA |
| *Il18* | CCTCTTGGCCCAGGAACAATG | ACAGTGAAGTCGGCCAAAGTT | *Tnfa* | ATGAGCACAGAAAGCATGATC | TACAGCCTTGTCACTCGAATT |
| *Il18r1* | CACAACGATCCTGAAAACAAGAGAT | AAGGTTCTCCCTCTACCACATGAA | *Zpb1* | CGCCAAGGCTCTGGGAATGA | TGTGTGACTCCAGAATGAGCTATGT |

Table S2: list of primers used for transcriptomic analysis (qRT-PCR and medium throughput)
